# Effect of linagliptin on glucose metabolism and pancreatic beta cell function in patients with persistent prediabetes after metformin and lifestyle

**DOI:** 10.1038/s41598-021-88108-8

**Published:** 2021-04-22

**Authors:** Mildred Fátima de la Luz Alvarez-Canales, Sara Stephania Salazar-López, Diana Farfán-Vázquez, Yosceline Estrella Martínez-López, Jessica Noemí González-Mena, Lilia Marisela Jiménez-Ceja, Katya Vargas-Ortiz, María Lola Evia-Viscarra, María Luisa Montes de Oca-Loyola, Franco Folli, Alberto Aguilar-García, Rodolfo Guardado-Mendoza

**Affiliations:** 1 Department of Medicine and Nutrition, University of Guanajuato, León, Guanajuato Mexico; 2 Department of Medical Sciences, University of Guanajuato, León, Guanajuato Mexico; 3 Endocrinology Department Hospital Regional de Alta Especialidad del Bajío, León, Guanajuato Mexico; 4 Endocrinology and Metabolism Dipartimento Di Scienze Della Salute, Universita’ Degli Studi Di Milano, Milan, Italy; 5 Asst Santi Paolo E Carlo, Milan, Italy; 6 Research Department Hospital Regional de Alta Especialidad del Bajío, Col. San Carlos La Roncha, Blvd.Milenio #130, 37660 León, Guanajuato Mexico

**Keywords:** Endocrinology, Endocrine system and metabolic diseases, Pre-diabetes

## Abstract

The goal of the study was to evaluate the effect of adding linagliptin to metformin and lifestyle on glucose levels and pancreatic β-cell function in patients with persistent impaired glucose tolerance (IGT) after 12 months of metformin and lifestyle. A single center parallel double-blind randomized clinical trial with 6 months of follow-up was performed in patients with persistent IGT after 12 months of treatment with metformin and lifestyle; patients were randomized to continue with metformin 850 mg twice daily (M group, n = 12) or linagliptin/metformin 2.5/850 mg twice daily (LM group, n = 19). Anthropometric measurements were obtained by standard methods and by bioelectrical impedance; glucose was measured by dry chemistry, insulin by chemiluminescence, and pancreatic β-cell function was calculated with the disposition index using glucose and insulin values during oral glucose tolerance test (OGTT) and adjusting by insulin sensitivity. The main outcomes were glucose levels during OGTT and pancreatic β-cell function. Patients in the LM group had a reduction in weight (−1.7 ± 0.6, p < 0.05) and body mass index (BMI, −0.67 ± 0.2, p < 0.05). Glucose levels significantly improved in LM group with a greater reduction in the area under the glucose curve during OGTT (AUCGluc_0_120min_) as compared to the M group (−4425 ± 871 vs −1116 ± 1104 mg/dl/120 min, p < 0.001). Pancreatic β-cell function measured with the disposition index, improved only in LM group (2.3 ± 0.23 vs 1.7 ± 0.27, p 0.001); these improvements persisted after controlling for OGTT glucose levels. The differences in pancreatic β-cell function persisted also after pairing groups for basal AUCGluc_0_120min_. The addition of linagliptin to patients with persistent IGT after 12 months of treatment with metformin and lifestyle, improved glucose levels during OGTT and pancreatic β-cell function after 6 months of treatment.

Trial registration: Clinicaltrials.gov with the ID number NCT04088461

## Introduction

Type 2 diabetes (T2D) is a chronic disease with pandemic characteristics^1^. In several countries like Mexico, T2D is one of the main causes of death, with an enormous economic impact, due mainly to micro and macrovascular complications, greatly impacting quality of life as well as lifespan^2–7^. Individuals with prediabetes, especially those with impaired glucose tolerance, are at high risk of developing T2D^8,9^.

Prediabetes is diagnosed by one of the following criteria: i) impaired fasting glucose (IFG) [fasting plasma glucose (FPG) ≥ 5.5 mmol/L [≥ 100 mg/dl]], (ii) impaired glucose tolerance(IGT) [2 h plasma glucose ≥ 7.8 mmol/L [140—199 mg/dl] after a 75-g glucose load in an oral glucose tolerance test (OGTT)], (iii) IFG + IGT, and/or by HbA1c (5.7–6.4%)^10,11^. Different pathophysiological abnormalities coexist in individuals with prediabetes, including pancreatic β-cell dysfunction, insulin resistance, reduced incretin effect and lipotoxicity, among others; with pancreatic β-cell dysfunction being a key factor involved in the progression of prediabetes to T2D^12–22^. The prevalence of prediabetes in different populations has been reported to be around 30%^23–25^. It is estimated that up to 70% of individuals with prediabetes eventually will develop T2D and its complications^4,26^.

Preventing/delaying the onset of T2D is becoming more important especially considering that patients with T2D may suffer from acute and chronic complications^27^. In fact, restoration of normoglycemia should be the main goal of any preventive strategy in T2D^28,29^. Different approaches have been employed to delay or prevent T2D, including lifestyle modification and pharmacotherapy mainly based on metformin^27,30–32^. However, lifestyle changes were insufficient to prevent T2D in ˃50% of cases due mainly to lack of adherence. Current guidelines recommend the use of metformin as a pharmacological treatment in patients with prediabetes together with lifestyle changes, although metformin reduces the risk of T2D only by 31%^30^.

Metformin is an antidiabetic drug that inhibits hepatic gluconeogenesis and reduces hepatic glucose output, increases glucose uptake and utilization in peripheral tissues (muscle and fat), and improves the energy metabolism in muscle, fat, and liver through the activation of AMP-activated protein kinase^33^. Dipeptidyl peptidase (DPP-4) inhibitors increase incretin hormone levels, which improve insulin secretion, insulin resistance and reduce glucagon levels^34,35^, and they have been proven to be effective and safe in patients with T2D^36–38^. Linagliptin is a DPP-IV inhibitor for T2D treatment, which is metabolized by the liver ^37,38^. Interestingly it improves beta cell function and reduces the risk of T2D onset in patients with prediabetes, when combined with metformin and a lifestyle modification program^39^. Considering these differential and complementary mechanism of action, we hypothesize that adding linagliptin to patients with prediabetes with scarce response to metformin would improve glucose metabolism and pancreatic β-cell function.

The goal of the present study was to evaluate the effect of adding linagliptin to metformin on glucose metabolism and β-cell function in patients with IGT not responding to metformin and lifestyle modification program during the previous 12 months.

## Materials and methods

The RESCATHEME project (**Resc**ue **A**fter **The**rapy with **Me**tformin) was a 6 month single center double-blind randomized clinical trial performed in patients with IGT who did not achieve normal glucose tolerance after one year of treatment with metformin and lifestyle modifications. Patients were recruited from a previous diabetes prevention study, performed at the Metabolic Research Laboratory in the University of Guanajuato, México, which evaluated the effect of metformin plus lifestyle modifications on T2D prevention for 12 months in patients with prediabetes^32^.

The protocol was approved by the Research and Ethical Committee at the Hospital Regional de Alta Especialidad del Bajío (CEI-35–16) and by the Ethical Committee at the University of Guanajuato (CIBIUG-P36-2016) and was registered at Clinicaltrials.gov with the ID number NCT04088461 (11/09/2019). All participants signed an informed consent form. All methods were carried out in accordance with the Research Guidelines by the National Health System, as well as in accordance with the International Research Guidelines and the Good Clinical Practice Standards.

## Participants

Eligibility criteria included patients of both sexes, 18–65 years of age, 2 h glucose levels between 140 and 199 mg/dl (7.8 to 11.0 mmol per liter) after a 75-g oral glucose load according to the criteria of the American Diabetes Association, despite being treated with metformin 850 mg twice daily plus lifestyle during the previous 12 months as a treatment for IGT. Patients were excluded from the study if they were taking medications, other than metformin, known to alter glucose metabolism during the previous 3 months, history of pathological conditions affecting glucose metabolism or body weight (thyroid disease, Cushing´s syndrome, Acromegaly), excessive alcohol intake (acute or chronic), pregnancy, systolic blood pressure > 180 mmHg or diastolic blood pressure > 105 mmHg (subjects could be re-screened after hypertension treatment), and T2D diagnosed during OGTT.

### Body composition and energy intake

Food consumption was evaluated by a semiquantitative food frequency questionnaire including data about the consumption of 116 food items; total energy intake was calculated by considering the energy intake from all foods^40^. Participants underwent anthropometric evaluation including measurements of body mass index (BMI), waist and hip circumference, and body composition evaluated by bioelectrical impedance employing a Tanita Scale SC-240.

### Metabolic evaluation

An OGTT was performed after an overnight fast of 8–10 h. An antecubital vein was cannulated to collect blood samples for determination of plasma glucose, insulin and lipid profile before ingestion of the 75-g glucose load. Blood samples were then drawn at 30, 60, 90, and 120 min for plasma glucose and insulin determinations.

### Clinical and biochemical measurements

Blood pressure was measured while the patient was at rest for at least 5–10 min with a mercury sphygmomanometer, and mean blood pressure (MBP) was calculated with the following: diastolic blood pressure (DBP) + (systolic blood pressure—DBP/3). Glucose was measured in duplicate with an Analox glucoanalyzer GM9 (Analox Instruments) and by colorimetric glucose oxidase (Vitros 5600; Ortho Clinical Diagnostics, CV 2.1–3.4%). Total cholesterol, HDL, LDL and triglycerides were measured in a single determination by dry chemistry with colorimetric method (Vitros 5600; Ortho Clinical Diagnostics). Insulin was measured in duplicates by a chemiluminiscent immunometric assay (IMMULITE 2000 Immunoassay system, Siemens, CV 3.3–5.5%). HbA1c was determined using high-performance liquid chromatography with a DS-5 Analyzer (Drew Scientific, Inc. Miami, FL, USA, CV 1.0–1.2%).

### Calculations

The incremental and the area under the glucose and insulin curve (*AUCglucose* and *AUCinsulin*) during the OGTT were calculated according to the trapezoidal rule. Insulin secretion was calculated dividing the *AUCinsulin*_*0_120*_ by the *AUCglucose*_*0_120*_ during the OGTT. Early phase of insulin secretion during OGTT was estimated by the insulinogenic index (IGI) as follows: Ins30-Ins0/Gluc30-Gluc0 (∆Ins30/∆Gluc30). Oral disposition index (DIo) was calculated by IGI × 1/fasting insulin^41^. The insulin secretion/insulin resistance (IS/IR) index (disposition index = DI) during OGTT was calculated as (*AUCinsulin*_*0_120*_/*AUCglucose*_*0_120*_)*Matsuda index^15^. Insulin sensitivity during OGTT was calculated from the Matsuda index^42^, and at fasting with the homeostasis model assessment (HOMA-IR).

### Randomization and masking

Patients were randomly assigned in a 1:1 ratio to receive linagliptin/metformin 2.5/850 mg every 12 h + lifestyle modification program (*LM group*), or to continue with metformin 850 mg every 12 h + lifestyle modification program (*M group*). Randomization was performed using an electronic random numbers assignment system by a Nutritionist who was not involved in the study. Participants and investigators involved in the patient’s follow-up and outcome measurements were masked to treatment allocation during the entire study duration by using identical envelopes for pills.

### Interventions

i) Linagliptin + metformin + lifestyle (*LM group*): Patients assigned to this group started combinations pills of linagliptin/metformin of 2.5/850 mg once daily during the first month, and after that the dose was increased to 2.5/850 mg twice daily from the second month until the end of the study. ii) Metformin + lifestyle (*M group*): Patients on this group continued with metformin 850 mg twice daily until the end of the study. Both groups received the same lifestyle modification program based on a prescribed diet every month, seeking to reduce their body weight at least by 5–7%, by adjusting their energy requirements based on their weight every two months. Diet was composed of 55–60% carbohydrates, 25–30% fat, and 10–15% proteins. If the patient was sedentary, he or she was advised to start with 45 min/week of mild to moderate exercise with a specific activity according to patient’s preference; patients were counseled to increase the duration and frequency or intensity of exercise every two weeks until reaching 150 min/week of moderate activity or 75 min/week of intense activity. If the patient was already physically active, it was recommended to continue like this and vary his/her exercise routines. Monitoring and modifications in diet and physical activity were performed at every appointment depending on each patient´s adherence to lifestyle modification. Food consumption was measured at baseline and after 6 months of intervention with a food frequency questionnaire. Physical activity was evaluated after 6 months of intervention with the long version of self-administered International Physical Activity Questionnaire^43^.

### Follow-up visits

Patients had a follow-up visit every month for 6 months. Every appointment was about 30–45 min; medications tolerance and side effects were recorded at every patient visit; nutritional assessment was performed by a Nutritionist every month. Monthly adherence to the medications was assessed based on pill counting.

### Statistical analysis and outcomes

Sample size calculation was based on an expected improvement of at least 60% in pancreatic β-cell function in patients with the combination of linagliptin + metformin, while in the metformin group β-cell function would improve by no more than 30%; it was also calculated considering a minimum difference of 3000 mg/dl/120 min in the AUCglucose during OGTT^39,44,45^. In order to be able to detect these differences between groups and assuming an alpha error of 5% and a beta error of 20%, we required a minimum of 12 patients per group.

The primary analysis was the comparison of the change in glucose profile during OGTT as well as measurements of pancreatic β-cell function during OGTT. Intergroup comparisons between absolute values and delta values were performed with a t-test for independent groups; intra-group comparisons were performed using a paired t-test. Repeated measures ANOVA was also used to evaluate inter and intra-group differences at -12, 0 and 6 months in the main outcomes. Non-numerical variables were compared between groups with the chi squared test. Statistical analyses and graphics were performed using SPSS Version 21.0 (SPSS Inc) and GraphPad Prism 5.0. Statistical significance was considered when p value was less than 0.05.

## Results

**Figure 1:**
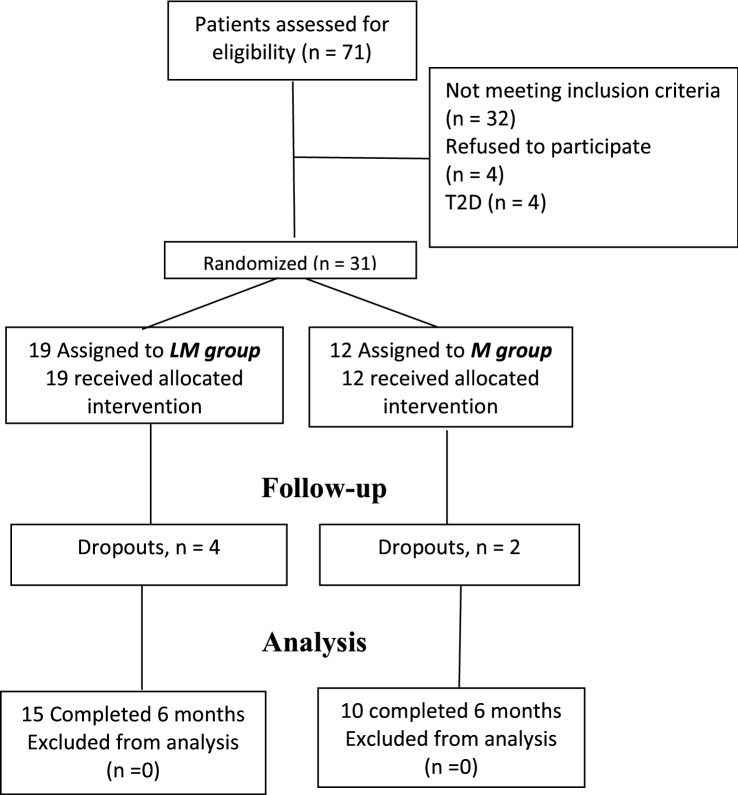
Study profile.

The study was performed between January 4, 2016, and August 30, 2018. A total of 71 patients were evaluated, but only 31 patients met the selection criteria and accepted to be enrolled in the study; 32 patients did not meet the selection criteria, 4 patients refused to participate in the study and 4 patients were diagnosed with T2D. Of the 31 patients enrolled in the study, 12 were randomly assigned to Metformin group (7 with isolated IGT and 5 with combined IFG + IGT) and 19 to linagliptin + metformin group (9 with isolated IGT and 10 with combined IFG + IGT); however, 2 patients in the metformin group and 4 in the linagliptin + metformin group were excluded from the study because they did not attend their follow-up appointments (Fig. 1). There were no differences in age or sex between the study groups (Table 1) as well as in the frequency of T2D risk factors with an average of 5 risk factors per patient (Suppl. Table 1).

**Table 1:** Clinical and anthropometric characteristics at the beginning and at the end of the study, in the treatment groups.

	Metformin			Linagliptin + Metformin		
	0 m (n = 12)	6 m (n = 10)	$\Delta$	0 m (n = 19)	6 m (n = 15)	$\Delta$
Sex (M/F)	2/12			6/13		
Age (years)	52 ± 2			53 ± 3		
MBP (mmHg)	91 ± 2	92 ± 3	1.2 ± 2.9	97 ± 3	93 ± 4^b^	−7.3 ± 3.2
Weight (Kg)	73 ± 4	76 ± 4	2.4 ± 2.7	75 ± 3	74 ± 3^b^	−1.7 ± 0.6
BMI (Kg/m^2^)	28.7 ± 1.6	29.8 ± 1.6	0.94 ± 1.0	29.2 ± 1.1	28.3 ± 1.2 ^b^	−0.67 ± 0.2
WC (cm)	92.4 ± 3.0	93.4 ± 3.7	−0.08 ± 3.5	93.0 ± 2.3	92.2 ± 3.1^b^	−3.6 ± 1.5
Body fat (%)	37.0 ± 2.3	37.2 ± 2.5	0.2 ± 1.3	35.3 ± 1.7	36.3 ± 2.0	0.3 ± 1.1
FPG (mg/dl)	96 ± 3	95 ± 2	−0.8 ± 3.0	103.0 ± 2.0	96 ± 1^b^	−9.1 ± 2.3^e^
Glucose 60 min (mg/dl)	167 ± 11	158 ± 18	−13 ± 12	194 ± 7^c^	144 ± 8^b^	−52 ± 12^e^
Glucose 120 min (mg/dl)	155 ± 4	141 ± 10	−10 ± 9	162 ± 6	124 ± 10^b^	−38 ± 10^e^
AUCglucose OGTT (mg/dl/120 min)	18,422 ± 755	17,395 ± 1476	−1116 ± 1104	20,283 ± 556	16,031 ± 710^b^	−4425 ± 871^e^
HbA1c % (mmol/mol)	5.4 ± 0.1(36 ± 1)	5.7 ± 0.1^a^(39 ± 1)	0.3 ± 0.1	5.6 ± 0.1^c^(38 ± 1)	5.5 ± 0.1^b^(37 ± 1)	−0.2 ± 0.1^e^
Cholesterol (mg/dL)	210 ± 6	189 ± 8	−18 ± 10	176 ± 6^c^	176 ± 8	−2 ± 6
HDLc (mg/dL)	48 ± 3	47 ± 4	1 ± 3	45.0 ± 3	54 ± 10	10 ± 10
Triglycerides (mg/dL)	176 ± 26	166 ± 15	8 ± 15	191 ± 27	169 ± 21	−47 ± 26

Data are Means ± SE.

$\Delta$= change from baseline, MBP Mean Blood Pressure, BMI body mass index, WC waist circumference. OGTT oral glucose tolerance test, AUC area under the curve, HbA1c: glycated hemoglobin A1c, HDL high-density lipoprotein cholesterol.

a = p < 0.05 Metformin intragroup.

b = p < 0.05 Linagliptin + Metformin intragroup.

c = p < 0.05 intergroup at the beginning of the study (0 months).

d = p < 0.05 intergroup at the end of the study (6 months).

e = p < 0.05 Delta intergroup at 6 months of study.

Before starting this study, patients had been treated with metformin for 12 months. No significant differences were observed in anthropometric, clinical, and biochemical variables between the study groups when they first started metformin (12 months before the current trial, Suppl. Tables 2, 3). No significant side effects were reported in both groups of treatment and the adherence to medications was higher than 80%.

After 6 months of follow-up during the current trial, only participants in the LM group had a significant reduction in body weight (−1.7 ± 0.6 kg, Suppl. Fig. 1) as well as mean blood pressure (−7.3 ± 3.2 mmHg), BMI (−0.67 ± 0.2 kg/cm^2^), and waist circumference (−3.6 ± 1.5 cm) (p < 0.05, Table 1); although these differences were not statistically significant when compared against the M group.

Baseline energy intake was 1714 ± 88 and 2084 ± 178 kcal/day in M and LM group, respectively (p = 0.129); after 6 months there was a reduction of around 15–20% in energy intake in both groups without differences between them (1323 ± 184 and 1785 ± 126 kcal/day in M and LM group, p = 0.137); this reduction was basically due to a reduction in carbohydrate intake (carbohydrates intake at baseline 271 ± 14 vs 318 ± 25 g/day, p = 0.168, and at 6 months 189 ± 32 vs 236 ± 21 g/day, p = 0.229, in M vs LM group, respectively), since fat intake was not different at baseline (50 ± 5 vs 48 ± 7 g/day, M and LM group, p = 0.727) and 6 months after intervention in both groups (49 ± 7 vs 52 ± 8 g/day, M and LM group, p = 0.781). Physical activity after 6 months of intervention was similar between groups (M 2791 ± 1230 vs LM 3260 ± 918 MET-min/week (p = 0.730), with 44% and 53% of patients with a moderate level of physical activity in M and LM group, respectively (p = 0.414).

**Figure 2:**
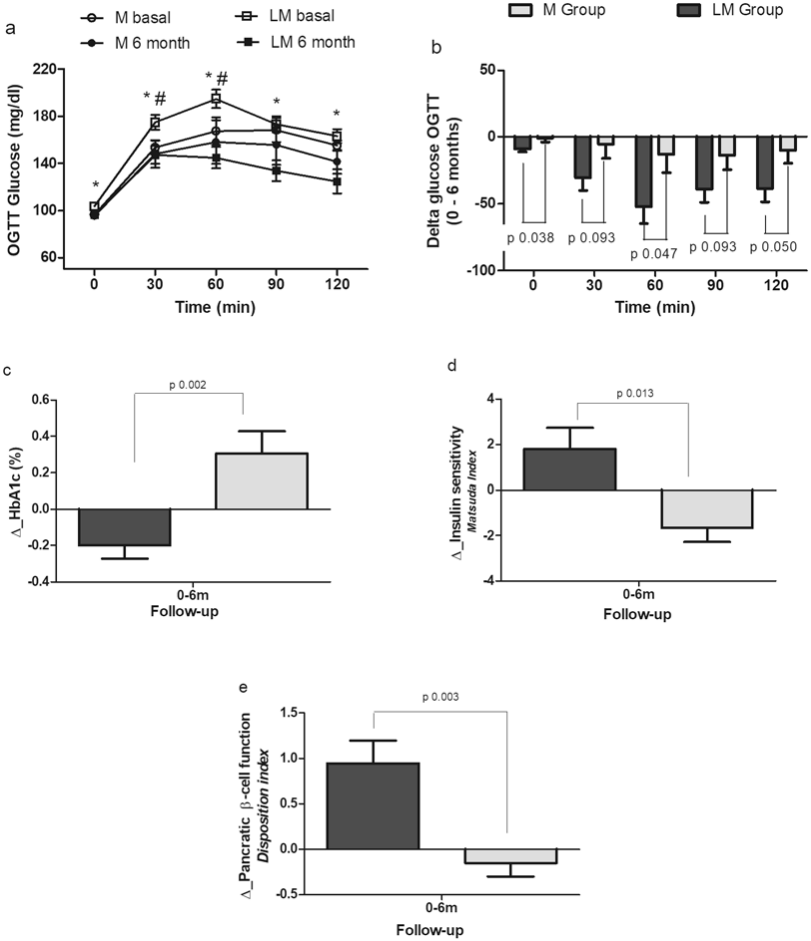
Glucose levels during OGTT at basal and after 6 months (**a**); change in glucose levels (**b**), HbA1c (**c**), insulin sensitivity (**d**), and pancreatic β-cell function (**e**) from 0 to 6 months between the study groups. * p˂0.05 for comparisons in LM group between basal and 6 months, # p˂0.05 for inter-group comparisons at basal (0 months).

The M group only had a small but no significant decrease in glucose levels during OGTT, while in the LM group there was a significant reduction in glucose levels during the whole OGTT (Fig. 2a,b, Table 1, p < 0.05 inter and intragroup). Moreover, glucose excursions were significantly reduced in LM group (AUCgluc_0_120min_ −4425 ± 871 vs −1116 ± 1104 mg/dl/120 min, p < 0.05; Suppl. Fig. 1b). These differences were also confirmed by two-way repeated measures ANOVA when including the three time points (−12, 0 and 6 months) in both groups (Suppl. Fig. 1b, M group p = 0.915, LM group p < 0.001). HbA1c was reduced in the LM group and significantly increased in M group [−0.283% (CI95% −0.487, −0.078), p = 0.011 vs + 0.358% (CI95% 0.059–0.656), p = 0.018, Suppl. Fig. 1c)] intergroup difference p < 0.05, Fig. 2c); 5 patients in the M group (50%) and 4 in the LM group (26.7%) had persistent IGT at the end of the 6 months of follow-up, and no cases of T2D were identified in both groups (p = 0.233). Lipid levels were not significantly different at the end of the study in the two treatment groups.

Insulin sensitivity measured by the Matsuda Index was significantly reduced in the M group, whereas it improved significantly in the LM group (from 4.2 to 5.3, p < 0.05; Fig. 2d, Table 2 and Suppl. Fig. 1d). Insulin secretion was increased only in the LM group, without reaching statistical significance (Suppl. Fig. 1e). Pancreatic beta-cell function, measured by the disposition index showed a significant improvement in the LM group by around 76% (1.3 to 2.3, p < 0.05), while it was slightly reduced in the M group (1.8 to 1.7) with a significant difference at the end of the study between the study groups (1.7 ± 0.2 vs 2.3 ± 0.2, p < 0.05) in favor of the LM group (Fig. 2e, Table 2 and Suppl. Fig. 1f), and this difference was also observed with the two-way repeated measures ANOVA including the three time points (−12, 0 and 6 months) with an increase of 0.982 (CI 95% 0.417–1.548, p = 0.010) in the disposition index in LM group, and not significant increase in M group (Suppl. Fig. 1f).

**Table 2:** Insulin resistance and insulin secretion at the beginning and end of the study.

	Metformin			Linagliptin + Metformin		
	0 m (n = 12)	6 m (n = 10)	$\Delta$	0 m (n = 19)	6 m (n = 15)	$\Delta$
Matsuda index	5.4 ± 1.1	5.3 ± 1.2^a^	−1.6 ± 0.6	4.2 ± 0.6^c^	5.3 ± 0.9^b^	1.8 ± 0.9^e^
HOMA – IR	2.0 ± 0.6	2.4 ± 0.5^a^	0.6 ± 0.2	2.6 ± 0.3	2.1 ± 0.3	−0.7 ± 0.3^e^
AIR (Ins30-Ins0/Gluc30-Gluc0)	1.1 ± 0.2	1.2 ± 0.3	0.27 ± 0.30	0.6 ± 0.1^c^	1.4 ± 0.4	0.70 ± 0.4
Insulin secretion (AUCins0-120/AUCgluc0-120)	0.46 ± 0.07	0.46 ± 0.10	0.09 ± 0.04	0.41 ± 0.04	0.54 ± 0.07	0.10 ± 0.05
Disposition Index (Matsuda*(AUCins_0-120_/AUCgluc_0-120_)	1.8 ± 0.1	1.7 ± 0.2	−0.15 ± 0.14	1.3 ± 0.1^c^	2.3 ± 0.2^d,b^	0.95 ± 0.24^e^
Oral Disposition Index (DIo) (IGI × 1/fasting insulin)	0.16 ± 0.02	0.14 ± 0.04	0.01 ± 0.03	0.07 ± 0.01^c^	0.19 ± 0.05^b^	0.10 ± 0.04

Data are Means ± SE. $\Delta$= change from baseline, AIR acute insulin response; AUCins0-120/AUCgluc0-120 Area under the insulin curve over the area under the glucose curve during OGTT, HOMA-IR Homeostasis Model Assessment, IGI insulinogenic index.

a = p < 0.05 Metformin intragroup.

b = p < 0.05 Linagliptin + Metformin intragroup.

c = p < 0.05 intergroup at the beginning of the study (0 months).

d = p < 0.05 intergroup at the end of the study (6 months).

e = p < 0.05 Delta intergroup at 6 months of study.

Since baseline glucose values were significantly higher in the LM group, we also performed a paired analysis to control for the confounding effect of glucose levels during OGTT at the beginning of the study (Table 3); 10 patients per group were paired by baseline AUCglucose and included for this analysis. Thus, even after adjusting by baseline glucose levels, there was a greater reduction in glucose levels during OGTT at 60, 120 min and HbA1c in the LM group at 6 months (Table 3, p < 0.05). Also, insulin sensitivity measured by Matsuda index had a decrease of −1.6 in the M group while the LM group showed an improvement of 1.1 (Table 3, p < 0.05), and pancreatic β-cell function measured with the disposition index, did not change in the M group, whereas it improved significantly in the LM group (Table 3, p < 0.05).

**Table 3:** Biochemical indicators before and after the intervention, in both groups after controlling for the baseline area under the glucose curve during OGTT.

	Metformin			Linagliptin + Metformin		
	0 m (n = 10)	6 m (n = 10)	$\Delta$	0 m (n = 10)	6 m (n = 10)	$\Delta$
FG (mg/dl)	96 ± 3	95 ± 2	−1 ± 3	102 ± 2	96 ± 2^b^	−7 ± 2
Glucose 120 min (mg/dl)	152 ± 5	141 ± 10	−10 ± 9	155 ± 9	122 ± 11^b^	−34 ± 12
AUCglucose OGTT (mg/dL/120 min)	18,511 ± 878	17,395 ± 1476	−1116 ± 1104	19,213 ± 387	15,666 ± 881^b^	−3547 ± 1094
HbA1c %	5.37 ± 0.12	5.68 ± 0.09^a^	0.30 ± 0.12	5.6 ± 0.1	5.49 ± 0.04	−0.18 ± 0.10^e^
Matsuda index	6.0 ± 1.3	4.4 ± 0.8^a^	−1.6 ± 0.6	3.8 ± 0.6	4.9 ± 0.9	1.1 ± 1.0^e^
HOMA – IR	1.9 ± 0.6	2.6 ± 0.6^a^	0.70 ± 0.26	2.5 ± 0.4	2.1 ± 0.4	−0.50 ± 0.28
AIR (Ins30-Ins0/Gluc30-Gluc0)	0.9 ± 0.2	1.2 ± 0.3	0.27 ± 0.30	0.8 ± 0.1	1.7 ± 0.5	0.93 ± 0.53
Insulin secretion (AUCins0-120/AUCgluc0-120)	0.39 ± 0.07	0.49 ± 0.11	0.09 ± 0.04	0.46 ± 0.05	0.54 ± 0.08	0.08 ± 0.06
Disposition Index (Matsuda*(AUCins0-120/AUCgluc0-120)	1.74 ± 0.14	1.59 ± 0.12	−0.15 ± 0.14	1.49 ± 0.10	2.20 ± 0.21^d,b^	0.71 ± 0.16^e^
Oral Disposition Index (DIo) (IGI × 1/fasting insulin)	0.14 ± 0.02	0.12 ± 0.04	0.01 ± 0.03	0.10 ± 0.01	0.22 ± 0.06	0.12 ± 0.05

Data are Means ± SE.

$\Delta$= change from baseline, AIR acute insulin response, OGTT oral glucose tolerance test, AUC area under the curve, HbA1c: glycated hemoglobin A1c.

a = p < 0.05 Metformin intragroup.

b = p < 0.05 Linagliptin + Metformin intragroup.

c = p < 0.05 intergroup at the beginning of the study (0 months).

d = p < 0.05 intergroup at the end of the study (6 months).

e = p < 0.05 Delta intergroup at 6 months of study.

## Discussion

In this small proof-of-concept study, we show that the addition of linagliptin to metformin during 6 months in patients with persistent IGT after 12 months of treatment with metformin and lifestyle changes, improves glucose levels during OGTT and pancreatic β-cell function in comparison to those patients who continue treatment only with metformin and lifestyle modification. The benefit of lifestyle and metformin in prediabetes has been previously demonstrated. The Diabetes Prevention Program study showed that lifestyle and metformin reduced the relative risk of T2D by 58 and 31%, respectively, after 2–3 years; however, it has been also observed a decline in the beneficial effect of these therapies and a progressive increase of T2D incidence have been observed^30,46^, possibly due to the nature of the disease or the lack of an effective impact on the physiopathology of hyperglycemia. Few studies have evaluated the effect of DPP-IV inhibitors in prediabetes, showing in general a beneficial effect^36,47–49^. We reported an improvement in glucose levels as well as in islet function in patients at high risk for developing T2D with the combination of linagliptin, metformin and lifestyle^39^. To our knowledge this is the first study to evaluate the additive effect of a DPP-IV inhibitor in patients with prediabetes previously treated with metformin and lifestyle and persistent impaired glucose tolerance^32^.

Lifestyle interventions, as well as pharmacological therapy with metformin, pioglitazone, GLP-1 receptor agonist, DPP-IV inhibitors, and the combination of them reduce glucose levels in patients with prediabetes^30,31,39,50–52^. In this study we found significant reductions in glucose levels as well as in HbA1c with the combination of linagliptin, metformin and lifestyle, which is relevant considering that these patients were previously treated with metformin and lifestyle with no improvements or deterioration in HbA1c.

Lifestyle programs have beneficial effects in weight control and glucose metabolism by improving insulin sensitivity, mainly in skeletal muscle, while metformin improves insulin sensitivity in liver, reducing hepatic gluconeogenesis, which could be beneficial mainly in subjects with impaired fasting glucose^30–32^. However, in patients with IGT insulin resistance occurs predominantly at the muscle level and have a more marked pancreatic β- and alpha-cell dysfunction together with a reduction in the incretin effect, requiring different interventions to improve glucose metabolism^16,53,54^. In our study, body weight, BMI and waist circumference were significantly reduced only in the LM group; although DPP-IV inhibitors have a neutral or slight effect on body weight, this could be due to the combination of lifestyle, metformin and linagliptin. Changes in mean blood pressure observed in LM group could be part of the vascular effects previously reported with incretin therapies by modulating renin-angiotensin system activity and improving endothelial dysfunction^55–57^.

DPP-IV inhibitors improve pancreatic islet function through the reduction in glucagon secretion and increase in insulin secretion^34,35,39^. In our study, we observed a significant improvement in pancreatic β-cell function with the combined treatment, which persisted after the groups were paired by basal glucose levels during OGTT, controlling in this way the confounding effect of different baseline glucose levels. This is consistent with previous studies with GLP-1 receptor agonist or DPP-IV inhibitors in prediabetes^39,47,51,52^. These findings point to the effect of DPP-IV inhibitors on preserving pancreatic β-cell function in prediabetes. These findings might also suggest a more proactive approach in the treatment of prediabetes not reverting to normoglycemia after a period of time with the standard treatment (metformin plus lifestyle modification), especially considering that pancreatic β-cell function is one of the main markers and prognostic factors involved in the progression to T2D^9,13^.

Insulin sensitivity was also significantly improved in the LM group, consistent with previous trials in prediabetes using incretin therapies^39,51,52^. Improvements in pancreatic β-cell function and insulin sensitivity might explain the reductions in glucose levels so that less patients in the LM group had persistent IGT in comparison to patients in the M group (n = 4, 26.7% vs n = 5, 50.0%, respectively).

All this together emphasizes the additive effect of combined therapies in physiopathology of prediabetes; therapies that may improve insulin sensitivity, like metformin and lifestyle, and therapies that can improve pancreatic islet function, like DPP-IV inhibitors, and highlights the better impact that these combined therapies may have in T2D prevention^39,51^.

No side effects were reported. Our study has several weaknesses; the small sample size and the short duration did not allow us to detect some other differences between the study groups that could be occurring, and that are just shown as trends. However, besides the small sample size we were able to see statistical differences mainly in glucose levels and pancreatic β-cell function, which were the main outcomes of the study. On the other hand, treatment groups were not homogenous at the beginning of this study, but this limitation was solved with the paired analysis where the differences in glucose and pancreatic β-cell function persisted. The interest of the study is that the population that was included had prediabetes already treated during 12 months with metformin and lifestyle, with persistent IGT. Thus, it seems that the effect of adding the DPP-IV inhibitor was still beneficial on glucose levels and pancreatic β-cell function in patients with prediabetes already treated with metformin and lifestyle.

## Conclusion

The addition of linagliptin in patients with persistent IGT after 12 months of treatment with lifestyle modification + metformin improves glucose levels during OGTT and pancreatic β-cell function. This approach should be evaluated in larger sample size population longer duration studies to identify more effective therapies to improve pancreatic β-cell function and reduce or delay T2D onset.

## Disclosure

The authors have nothing to disclose. All authors have approved the final version of the manuscript.

## Supplementary Information

Supplementary Information

## Data Availability

The datasets generated during and analyzed during the current study are not publicly available but are available from the corresponding author on reasonable request.
