# Supplementary material for: Effect of linagliptin on glucose metabolism and pancreatic beta cell function in patients with persistent prediabetes after metformin and lifestyle
Source: Sci Rep. 2021 Apr 22;11:8750. doi: 10.1038/s41598-021-88108-8 (PMC8062549; doi:10.1038/s41598-021-88108-8)
Supplement: Supplementary file 1 — Supplementary Information [file 41598_2021_88108_MOESM1_ESM.pdf]

# Effect of Linagliptin on Glucose Metabolism and Pancreatic Beta Cell Function in Patients with Persistent Prediabetes After Metformin and Lifestyle

Mildred Fátima de la Luz Alvarez-Canales MS*, Sara Stephania Salazar-López MD*, Diana Farfán-Vázquez MD, Yosceline Estrella Martínez-López CN MS, Jessica Noemí González-Mena CN MS, Lilia Marisela Jiménez-Ceja MD PhD, Katya Vargas-Ortiz PhD, María Lola Evia-Viscarra MD, María Luisa Montes de Oca-Loyola MD, Franco Folli MD PhD, Alberto Aguilar-García MD, Rodolfo Guardado-Mendoza MD PhD

**Mildred Fátima de la Luz Alvarez-Canales and Sara Stephania Salazar-López contributed equally to this article*

Mildred Fátima de la Luz Álvarez-Canales MS, Department of Medicine and Nutrition University of Guanajuato, León, Guanajuato, México nutr_mac@yahoo.com

Sara Stephania Salazar-López MD, Department of Medicine and Nutrition University of Guanajuato, León, Guanajuato, México sarastephaniasalazarlopez@gmail.com

Diana Farfán Vázquez MD, Department of Medicine and Nutrition University of Guanajuato, León, Guanajuato, México yopli_diana@hotmail.com

Yoscelina Estrella Martínez-López MS, Department of Medicine and Nutrition University of Guanajuato, León, Guanajuato, México ruxlia@hotmail.com

Jessica Noemí González-Mena, Department of Medicine and Nutrition University of Guanajuato, León, Guanajuato, México jessica.mena27@gmail.com

Lilia M. Jiménez-Ceja MD PhD, Department of Medicine and Nutrition University of Guanajuato, León, Guanajuato, México lilicolima@hotmail.com

Katya Vargas-Ortiz, Department of Medical Sciences, University of Guanajuato, León, Guanajuato, México kavati75@hotmail.com

Maria Lola Evia-Viscarra MD, Endocrinology Department Hospital Regional de Alta Especialidad del Bajío, León, Guanajuato, México evialola@hotmail.com

María Luisa Montes de Oca-Loyola MD, Department of Medicine and Nutrition University of Guanajuato, León, Guanajuato, México mariamol_92@hotmail.com

Franco Folli MD PhD, Endocrinology and Metabolism Dipartimento di Scienze della Salute Universita' degli Studi di Milano, franco.folli@unimi.it. Asst Santi Paolo E Carlo, Millano, Italy franco.folli@asst-santipaolocarlo.it

Alberto Aguilar-García MD, Endocrinology Department Hospital Regional de Alta Especialidad del Bajío, León, Guanajuato, México betaag@yahoo.com.mx

Rodolfo Guardado-Mendoza MD PhD, Research Department Hospital Regional de Alta Especialidad del Bajío and Department of Medicine and Nutrition University of Guanajuato, León, Guanajuato, México guardamen@gmail.com, rguardado@ugto.mx

**Corresponding author**: Rodolfo Guardado-Mendoza MD PhD, guardamen@gmail.com , Blvd.Milenio #130, Col. San Carlos la Roncha, León Guanajuato. CP. 37660, Tel.+52 (477) 267 2000.

Suppl. Table 1. Prevalence of T2D risk factors between the study groups

| n = 31 | M group (n=12) | LM group (n=19) | p value |
|---|---|---|---|
| # T2D risk factors | 5 (5-6) | 5 (5-6) | 0.809* |
| T2D risk factors, n (%) | | | |
| Ethnicity | 12 (100) | 19 (100) | 1.000 |
| Physical inactive | 7 (58.3) | 11 (57·9) | 0.981 |
| Age >45 years | 10 (83·3) | 15 (78.9) | 0.574 |
| T2D family history | 9 (75.0) | 18 (94.7) | 0·272 |
| HBP | 2 (16.7) | 4 (21.1) | 0·574 |
| Birth baby >4 kg | 1 (8.3) | 0 (0) | 0.387 |
| POS | 1 (8.3) | 0 (0) | 0·387 |
| BMI >27 kg/m$^2$ | 8 (66.7) | 12 (63.2) | 0·842 |
| Acanthosis nigricans | 1 (8.3) | 3 (15.8) | 0·546 |
| Dyslipidemia | 11 (91.6) | 17 (89.4) | 0·426 |
| History of CVD | 0 (0) | 0 (0) | |

*Mann-Whitney U test; median (Q1-Q3). T2D: type 2 diabetes, HBP: high blood pressure, POS: polycystic ovary syndrome; BMI: body mass index; CVD: cardiovascular disease.

Suppl. Table 2. Anthropometric and clinical variables 12 months before the start of this study, in both treatment groups

| | Metformin | Linagliptin + Metformin | |
|---|---|---|---|
| | -12 m (n=12) | -12 m (n=19) | P |
| Sex (M/F) | 2/12 | 6/13 | 0.355 |
| Age (years) | 51 ± 2 | 52 ± 2 | 0.202 |
| MBP (mmHg) | 93 ± 3 | 97 ± 3 | 0.986 |
| Weight (Kg) | 75 ± 4 | 77 ± 3 | 0.724 |
| BMI (Kg/m$^2$) | 29.7 ± 1.6 | 30.0 ± 1.2 | 0.601 |
| WC (cm) | 90 ± 3 | 94 ± 3 | 0.618 |
| Body fat (%) | 37.5 ± 2.3 | 35.8 ± 1.9 | 0.565 |
| Visceral fat (au) | 10.0 ± 1.1 | 10.7 ± 0.9 | 1.000 |
| FG (mg/dl) | 102 ± 2 | 105 ± 2 | 0.161 |
| Glucose 60 min (mg/dl) | 168 ± 12 | 175 ± 7 | 0.940 |
| Glucose 120 min (mg/dl) | 157 ± 6 | 147 ± 7 | 0.256 |
| AUCglucose OGTT (mg/dl/120 min) | 18601 ± 1036 | 18932 ± 612 | 0.998 |
| HbA1c % (mmol/mol) | 5.3 ± 0.2 (34 ± 2) | 5.2 ± 0.1 (33 ± 1) | 0.259 |
| Cholesterol (mg/dL) | 212 ± 12 | 196 ± 8 | 0.991 |
| HDLc (mg/dL) | 49 ± 3 | 43 ± 3 | 0.228 |
| LDLc (mg/dL) | 132 ± 13 | 122 ± 7 | 0.826 |
| Triglycerides (mg/dL) | 165 ± 20 | 176 ± 24 | 0.524 |

Mean ± SE. MBP Mean Blood Pressure BMI body mass index, WC waist circumference, OGTT oral glucose tolerance test, HbA1c: glycated hemoglobin A1c, AUC area under the curve, HDL high-density lipoprotein cholesterol, LDL low density lipoprotein cholesterol.

Suppl. Table 3. Insulin resistance and pancreatic β-cell function 12 months before the start of this study in both treatment groups.

| | Metformin -12 m (n=12) | Linagliptin + Metformin -12 m (n=19) | p |
|---|---|---|---|
| Matsuda index | 4.1 ± 0.7 | 3.9 ± 0.4 | 0.857 |
| Acute Insulin Response (AIR_Ins30-Ins0/Gluc30-Gluc0) | 1.2 ± 0.2 | 1.0 ± 0.2 | 0.832 |
| Insulin secretion (AUCins0-120/AUCgluc0-120) | 0.45 ± 0.09 | 0.45 ± 0.06 | 0.823 |
| Disposition Index (Matsuda*(AUCins0-120/AUCgluc0-120) | 1.3 ± 0.1 | 1.4 ± 0.1 | 0.337 |
| Oral Disposition Index (DIo) (IGI×1/fasting insulin) | 0.13 ± 0.05 | 0.11 ± 0.02 | 0.455 |

Data are Means ± SE. AUCins0-120/AUCgluc0-120 Area under the insulin curve over the area under the glucose curve during OGTT, IGI insulinogenic index.

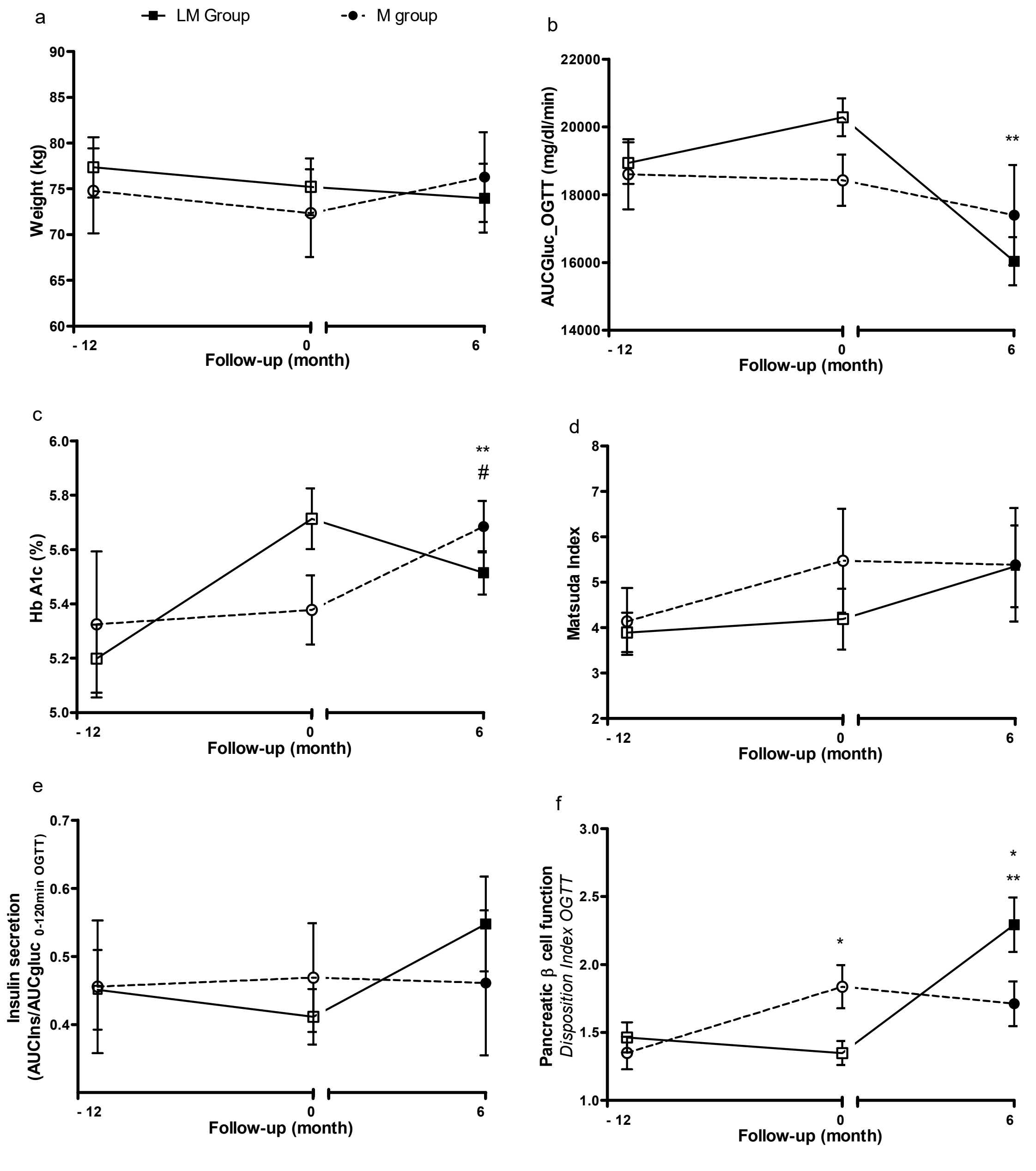


Suppl. Figure 1. Weight (a), $AUCGluc_{0\_120min}$ (b), HbA1c (c), Insulin sensitivity (d), insulin secretion (e), and pancreatic β-cell function (f) in both groups of treatment during the 12 previous months of the study, and after 6 months of follow-up during the present study. *$p<0.05$ inter-group at the specific time; ** $p<0.05$ for comparisons in LM group between 0 and 6 months; # $p<0.05$ for comparisons in M group between 0 and 6 months.
